# Autoimmune limbic encephalitis: A manifestation of systemic lupus erythematosus in the central nervous system

**DOI:** 10.1590/1980-57642015DN92000014

**Published:** 2015

**Authors:** Débora Bartzen Moraes Angst, Nathália Stela Visoná de Figueiredo, Valmir Passarelli, Meire Argentoni Baldocchi, Maria Sheila Guimarães Rocha, Sonia Maria Dozzi Brucki

**Affiliations:** Department of Neurology, Hospital Santa Marcelina, São Paulo SP, Brazil.

**Keywords:** limbic encephalitis, lupus erythematosus systemic, neoplasms

## Abstract

Autoimmune limbic encephalitis (ALE) associated with systemic lupus erythematosus (SLE) is a rare entity with few reports in the literature to date. In general, ALE associated with SLE has a satisfactory response to immunosuppressive treatment (RIT), but the pathogenesis of this association is poorly understood and may include an autoimmunity component. We report a case study describing the diagnosis and management of limbic encephalitis in a patient with active Systemic Lupus Erythematosus disease (SLE) and past medical history of cancer (endometrial adenocarcinoma in 2004 and papillary urothelial carcinoma in 2011 with curative treatment), followed over a one-year period. We discuss the possible association between limbic encephalitis and all past neoplastic and immune-mediated conditions of this patient. In this particularly case, autoimmunity was the most relevant factor associated with limbic encephalitis given negative neoplastic screening. Moreover, a good response was observed to immunotherapy, not seen with paraneoplastic limbic encephalitis, which is associated with poor response. In this case, the association of ALE with SLE is possible, since laboratory testing disclosed lupic activity and the patient had involvement of other systems (such as hematologic) during the period. However, the presence of other surface membrane antibodies are possible in the search for alternative etiologies.

## Abstract (pt)

Encefalite Límbica Autoimune (EL) associada a lúpus eritematoso sistêmico (LES) é uma entidade rara, com poucos relatos na literatura até o momento. Em geral, EL associada com LES tem uma resposta satisfatória ao tratamento imunossupressor, mas a patogênese desta associação é pouco compreendida e pode incluir um componente de autoimunidade. Descrevemos em um estudo de caso o diagnóstico e o tratamento empregado na encefalite límbica ocorrida no contexto de uma paciente com LES ativo e história pregressa de doenças neoplásicas (adenocarcinoma endometrial em 2004 e carcinoma papilar urotelial em 2011 ambos com o tratamento curativo), a qual foi seguida durante um ano. Discutimos uma possível associação de encefalite límbica e todos os antecedentes neoplásicos e imunomediados desta paciente. Neste caso em particular, a autoimunidade é o fator mais relevante relacionado com a encefalite límbica devido a uma triagem neoplásica negativa. Além disso, houve uma grande resposta com a imunossupressão, o que não é visto na encefalite límbica paraneoplásica, mais relacionada com uma má resposta. Neste caso, a associação de EL com LES é possível, uma vez que testes laboratoriais confirmaram a atividade lúpica, bem como a paciente apresentava envolvimento de outros sistemas (como hematológico) neste interim. No entanto, a presença de outros anticorpos de superfície da membrana é possível em busca de diferentes etiologias.

## INTRODUCTION

Limbic encephalitis (LE) is a rare neurological syndrome that selectively affects the structures of the limbic system.^1^ The main clinical manifestations of limbic encephalitis are seizures associated with episodic memory impairment and behavioral changes. In addition, there may be different degrees of involvement in extra-limbic-system tissues such as the cerebellum, brainstem and thalamus.^1,2^

In 1960, Brierley et al. first referred to the entity which affects the limbic areas as 'subacute encephalitis'.^3,4^ The disease was given its final name of 'limbic encephalitis' in1968 by Corsellis et al.^5,6^ Initial reports of this disease were accompanied by a positive history of cancer in the clinical context.^7^ Subsequent investigations confirmed this initially reported association and, based on substantial evidence, it was referred to as a classical paraneoplastic syndrome.^8^

Consequently, up until the mid‐1990s, most cases of LE were considered to be paraneoplastic.^9,10^ However, there is a growing number of reports of patients whose clinical, radiological and CSF findings suggest a clinical picture of limbic encephalitis, with both diagnostic tests and follow‐up excluding an underlying cancer.^11^ For this reason, the concept of limbic encephalitis has now been expanded. Although it is still considered a classical paraneoplastic syndrome, its association with autoimmune disease has been extensively studied.^12,13^

The discovery of these autoimmune disorders has changed the diagnostic approach to clinical problems as diverse as catatonia, subacute memory disturbance, as well as limbic encephalitis. For instance, some patients previously thought to have viral encephalitis will be found to have a treatable autoimmune disease.^13^ The incidence of these disorders related with an autoimmune mechanism is unknown, but collectively they are at least 5 times more frequent than all encephalitis cases associated with classic paraneoplastic antibodies.^13^

The association of autoimmune limbic encephalitis (ALE) and Systemic Lupus Erythematosus (SLE) has been recently highlighted.^14-17^ However, few articles have described this feature. Therefore, there is a lack of understanding on the frequency and power of this association.^16^

We report a case study, followed up for a one-year period, of a patient with limbic encephalitis with active Systemic Lupus Erythematosus Disease (SLE), who showed a good response to immunosuppressors and whose diagnostic tests excluded underlying active cancer.

## CASE REPORT

A right-handed 44-year-old female patient with 15 years of schooling was admitted in early February/2014 to our service with a history of asthenia and myalgia which started 7-10 days prior to admission. These symptoms were followed by anterograde amnesia and temporal disorientation initiated 3 days before the hospitalization. Clinical and neurologic examination was normal except for temporal disorientation, low scores on the Mini-Mental State Examination and episodic memory impairment (Table 1).

**Table 1:** Cognitive performance on baseline and follow-up.

Cognitive Assessment Follow-up							
		Baseline	10 d after PT	60 d	90 d	180 d	220 d
MMSE		22	27	30	30	30	30
BBRC	• Naming	10	10	10	-	10	10
	• Perception	10	10	10	-	10	10
	• Incidental memory	4	6	7	-	6	9
	• Immediate memory	4	7	8	-	8	10
	• Learning	7	7	9	-	9	10
	• Delayed recall	2	5	7	-	8	10
	• Recognition	3	8	10	16	10	10
Clock drawing		10	10	10	10	10	10
Semantic fluency		12	19	15	18	18	22
Phonemic fluency		17	19	16	-	-	-

d: days; PT: pulsotherapy; MMSE: mini mental status examination; BBRC: brief battery; -: not shown.

The patient reported previous diagnosis of SLE as well as endometrial adenocarcinoma in 2004 and papillary urothelial carcinoma in 2011, with a curative treatment in the past, and no complaints related to these diseases.

**Figure 1:**
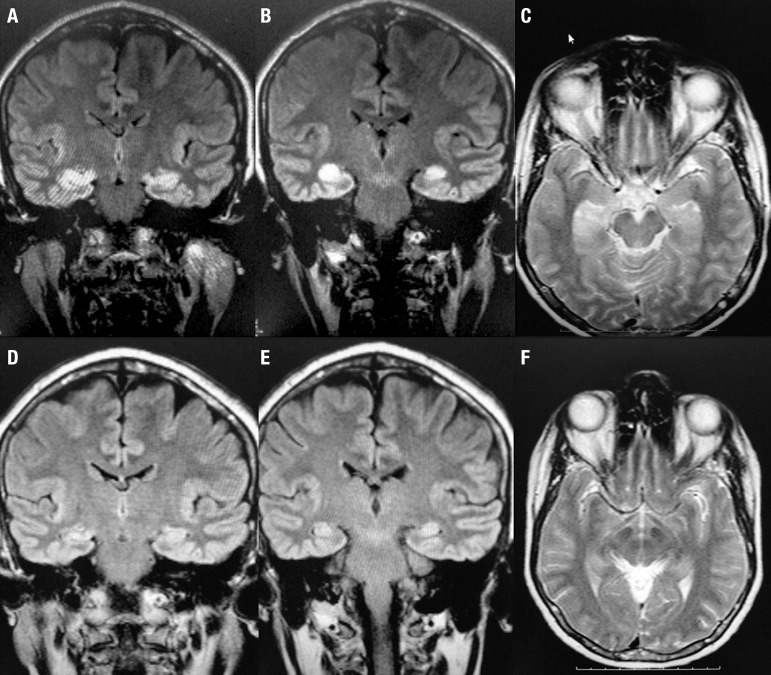
[A, B] Coronal flair, [C] Axial T2WI. [A, B] Bilateral hipocampi hyperintense sign at clinical onset of symptoms. [C] The same signal is already present on axial T2 sequence. [D, E] Coronal flair, [F] Axial T2Wi. [D, E] Later in the follow up (220 days after), MRI has an improvement. [F] The patient's total recovery is showed.

**Figure 2:**
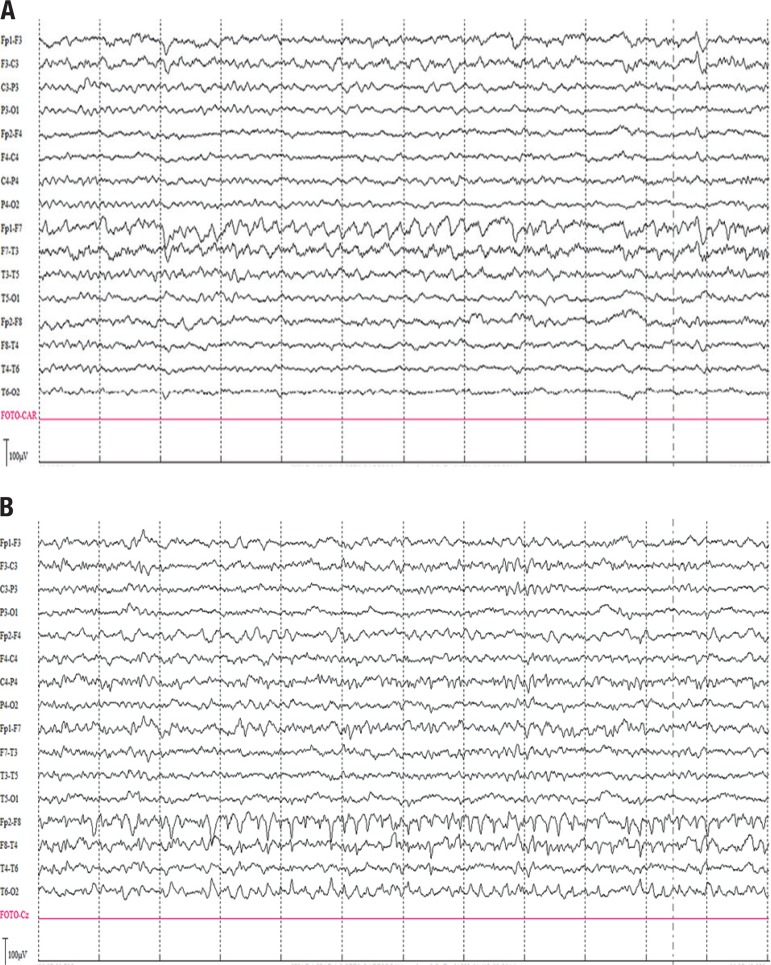
[A] EEG at clinical onset of symptoms has a TIRDA pattern at left temporal. [B] Through the same EEG, a seizure at right temporal is also register. In the follow up, it became normal without epileptic activity (not shown).

Her Magnetic Resonance Image (MRI) disclosed bilateral hippocampi hyperintense signal on T2 and Flair with restriction in diffusion and absence of abnormalities in ADC at admission (Figure 1). Moreover, CSF had mild lymphocytic pleocytosis (5 cells), 36.3 mg/dL of protein and 56 glucose. Her electroencephalography revealed a TIRDA pattern in the left temporal region with an electrographic seizure in the right temporal region on the same exam (Figure 2).

Extensive laboratory work-up was performed and serum assays showed low C3 and C4 complement fraction, presence of anti-P ribosomal, positive anti SSA (276ua/ml) and ANA (1/160) as well as lymphopenia and thrombocytopenia, clear signs of active SLE. A diagnosis of limbic encephalitis and active SLE was then reached.

Additionally, during the hospital stay, a search for tumors was performed. A laboratory study with biomarkers showed carcinoembryonic antigen, CA15-3, CA125 and CA19/9 at normal levels. Computed Tomographic imaging of the thorax, abdomen and pelvis, breast and transvaginal ultrasound were normal. MRI of the thorax, abdomen and pelvis was also performed and were normal. In addition, a Positron Emission Tomography (PET-CT) scan of the whole body was negative for any occult neoplastic focus.

Immunosuppressive treatment (IT) with methylprednisolone (1 g/d for 4 days) and cyclophosphamide 1g - single dose) was indicated. Video-electroencephalography was performed on the third day of immunosuppressive treatment, while using oxcarbazepine, and showed normal background activity without epileptic discharges. After this first session, at the end of February/2014, the patient improved her scores on learning, delayed recall and recognition. Her semantic and phonemic fluency were substantially increased. As clinical response was effective, it was decided to maintain the patient under this treatment regimen for at least 6 months to consolidate immunosuppression, with monthly infusions administered in the hospital setting. She was treated from February to October/2014.

During the follow-up, after two cycles of immunosuppression, a repeat MRI (April/2014) revealed a marked improvement in hippocampal signal on T2 and Flair compared to that observed in the first exam. Another MRI exam was performed in October/2014 (9 months from first admission) with normal signal in the hippocampi. A follow-up EEG remained normal, therefore the antiepileptic drug was discontinued. A fully and persistent recovery of her cognitive abilities was observed (Table 1) up until her last evaluation. Currently, she has a subjective complaint of slow thinking and normal instrumental activities of daily living. After concluding 6 months of immunosuppression treatment, she returned to work.

## DISCUSSION

The classical manifestation of LE includes episodic memory impairment^9^, seizures^10^, confusion^16^, sleeping problems^18^, and psychiatric symptoms.^19^ The most characteristic clinical feature is short-term memory loss, but associated symptoms such as confusion and seizures might limit the memory assessment.^12^

Gultekin et al. proposed diagnostic criteria that includes a pathological demonstration of LE or all of the following four:

[1] short-memory loss, seizures or psychiatric symptoms suggestive of limbic system involvement;
[2] less than 4 years between the neurologic symptoms and cancer diagnosis;
[3] exclusion of metastasis, infection, metabolic and nutritional deficits, stroke and side-effects of therapy that can cause LE;
[4] at least one out of: CSF with inflammatory findings; or hyperintensity of temporal lobes bilaterally on magnetic resonance image (MRI) T2/FLAIR sequences; or EEG with epileptic / slow activity involving focally temporal lobes^35^.

Other authors, Graus and Saiz, revised the criteria, changing some accepted clinical characteristics for diagnosis (under this criteria, the patient has to have all four items):

[1] subacute onset (less than 12 weeks) of the clinical signals and symptoms cited above;
[2] neuropathologic or radiologic (MRI, or single photon-emission computer tomography (SPECT); positron-emission computed tomography (PET-CT) evidence of limbic involvement;
[3] exclusion of other possible etiologies;
[4] demonstration of cancer within 5 years of the neurologic symptoms or the evidence of well-characterized paraneoplastic antibodies associated with this clinical picture^36^.

All patients with LE should undergo a neuroimaging evaluation of the medial temporal area.^1^ In patients with predominant *anterograde amnesia*, MRI usually discloses FLAIR or T2 abnormalities in this area.^1^ In individuals with a wide range of symptoms, the MRI shows more extensive abnormalities in the temporal lobes or beyond the limbic system.^1,20^ EEG often demonstrates unilateral or bilateral temporal lobe epileptic discharges or slow background activity.^12^ However, LE can present as an unexplained subacute onset of neurological symptoms, with normal MRI and no cerebrospinal fluid (CSF) evidence of inflammation.^11^

It is crucial to rule out any underlying malignancy as LE is commonly related to neoplasm, as a paraneoplastic manifestation. Therefore, the most frequent associated tumors are lung (particularly small cell lung cancer - SCLC), breast, ovarian, testicular, and prostate cancer and can be associated with thymoma, neuroendocrine tumors or Hodgkin´s disease.^12-14^ To our knowledge, only a single study has identified a case of limbic encephalitis, with positive VGKC antibodies associated with endometrial adenocarcinoma, whereas no reports of papillary urothelial carcinoma and limbic encephalitis were found.^21^

Thus, Fluorodeoxyglucose-PET is useful for detecting many occult malignancies but has limited utility for ovarian teratomas.^11^ For this type of tumor, MRI of the abdomen and pelvis is the test of choice, followed by CT and abdominal or transvaginal ultrasound (if age-appropriate).^11,13^ It is also important to order tests of tumor markers such as CA125, human chorionic gonadotropin, and alpha-fetoprotein.^11,13^

On the other hand, a few reports had previously described a possible association between LE and SLE. In this context, SLE for instance can mimic or be associated with limbic encephalitis.^15,22,23^ As lupus can present in many forms, it has been called 'the disease with a thousand faces'.^11,17,23-25^ The disturbances in neuropsychiatric SLE are wide-ranging and include cerebrovascular disease, seizures, myelopathy, aseptic meningitis, movement disorders, demyelinating syndrome as well as moderate or severe cognitive dysfunction, psychosis, acute confusional state and depression.^15^

Although autoimmune limbic encephalitis (ALE) and paraneoplastic limbic encephalitis (PLE) share an immune-mediated background, they can be separated as distinct medical conditions since they have different pathogenic mechanisms. Moreover, limbic encephalitis with active SLE, although with a poorly understood pathophysiology, could also share some similarities and differences in this picture. It is believed that a convergence point between the three conditions involves an autoimmunity component shared by all of them.^16^

In general, several studies show that five features characterize autoimmune physiopathology.^13,18,26^ Firstly, the epitopes are extracellular and the antibody binding is visible in cells transfected with the target antigen. Secondly, the antibodies alter the structure or function of the corresponding neuronal antigen. Thirdly, the effects of the antibodies are often reversible. Lastly, the clinical picture resembles that of pharmacologic or genetic models in which the antigen is disrupted.^13,18,26^

Specifically in the context of ALE and PLE, some researchers propose that a logical way to differentiate these two conditions is to identify whether the target antigen is intracellular, synaptic or on the cell surface and whether the immune response is primarily mediated by cellular or humoral mechanisms.^11^ Many studies indicate that the disorders associated with antibodies against intracellular antigens are mediated by T-cell mechanisms, which represent markers of an associated cancer but have not been shown to be pathogenic.^12,18,27,28^ These typically affect older individuals. Moreover, they are paraneoplastic and largely resistant to immunotherapy, even after tumor removal.^16,29,30^

In contrast, autoimmune limbic encephalitis is associated with antibodies to synaptic or cell surface antigens. These are likely to contribute directly to the pathology of the condition.^10,11^ They affect younger individuals as well as children and are often not associated with tumors. This condition appears to be antibody mediated, and is often highly responsive to treatment.^11,12^ Some immune-mediated cases appear to have a monophasic course, but others may relapse.^11^ The main antibodies related to nonparaneoplastic autoimmune LE are against NMDA receptors and VGKC complex.^31^

Therefore, with regard to ALE and PLE, there are many types of antibodies against extra or intracellular structures, such as: α-amino-3-hydroxy-5-methyl-4- isoxazolepropionic acid receptors (AMPARs), γ-aminobutyric acid-B receptors (GABABRs), glutamic acid decarboxylase (GAD), N-methyl-D-aspartate receptor (NMDAR) and voltage-gated potassium channel (VGKC) complex antigens: leucine rich glioma inactivated protein 1 (LGI1), and contactin-associated protein-2 (CASPR2). Additionally, there are onconeural antibodies, particularly anti-Hu, anti-Ma 1/2, CV-2, and amphiphysin.^11,32-34^

Specifically in SLE, some autoantibodies such as anti-phospholipids (β2-glycoprotein 1 and cardiolipin), anti-ribosomal P protein, anti-NMDA, specifically subtype Glun2 or NR2, and anti-microtubule-associated protein 2 (MAP-2) are found but with variable frequency in neuropsychiatric SLE.^15^ Besides all these, anti-glutamate receptor may be found to link these two diseases.^14^

Recent data suggests that neuropsychiatric events occur in 6–12% of patients with newly diagnosed SLE during the first year of the illness. The most common neuropsychiatric syndromes attributed to SLE are seizure disorders, cerebrovascular disease, acute confusional states and neuropathies.^37^

However, general SLE-related disease activity, previous or concurrent neuropsychiatric symptoms, and persistent positivity for antiphospholipid antibodies at moderate-to-high titers have been shown to be the most informative indicators of neuropsychiatric events attributed to SLE.^15^

For instance, the presence of anti-NMDA, specifically the subtype Glun2 or NR2, in patients with SLE is estimated at 14 to 37% and especially SLE patients with neuropsychiatric manifestations this figure can reach up to 80%.^14^ This association is a recent finding in the literature, and many authors are focusing on this previously unknown association. However, more elevated levels of anti-NMDA subtype Glun1 or NR1 are more frequently associated with Anti-NMDA receptor Encephalitis and seem to play a more evident pathological role in LE and may be dose-dependent.^14,15^

Consequently, irrespective of age, previous medical history and main presentation, ideally all such patients should be tested for these types of antibodies. Unfortunately, these antibodies in our patient could not be tested due to technical laboratory limitations.

In the management of ALE, it is important to highlight the benefits of early treatment^11^. General concepts about treatment of classical paraneoplastic CNS syndromes do not apply in these cases. For example, whereas classical paraneoplastic syndromes do not respond to immunotherapy unless the tumor is successfully treated^12^, when it then has a limited response^11,16,21^, ALE may respond to immunotherapy regardless of tumor removal.^12,13,26^

In conclusion, the primary objective of this article was to explore the possibility of an association between limbic encephalitis and autoimmune disease, particularly SLE. There is growing interest in the literature to study a possible association between autoimmune diseases, such as SLE, and limbic encephalitis, not thought possible only a few years ago. Knowledge about this clinical association and the pathophysiological mechanisms involved needs to be furthered. This article intends to contribute by reporting a possible case of LE with active SLE, adding more data to this discussion.

The main limitation of this article is the absence of neuronal antibody tests. However, the good evolution of the patient and excellent, rapid response to immunotherapy make it reasonable to assume an underlying autoimmune LE, as this has been discussed previously in the literature.^37^ By contrast, paraneoplastic LE usually has a poor response. The association of ALE with SLE is also reasonable, since laboratory testing showed SLE activity besides involvement of other systems (hematologic) concomitant to CNS involvement.

The presence of other surface membrane antibodies is possible, since there are associations among different types of these as mentioned above. This case highlighted a need for rapid treatment when an autoimmune cause is suspected.

To sum up, further studies are necessary to determine the true association between limbic encephalitis and autoimmune diseases, especially Systemic Lupus Erythematosus. Efforts should be made to establish whether this association is pathologic. Future studies should explore which antibodies are related to neuropsychiatric lupus and the pathologic mechanism triggered by them. To this end, determining the role of autoantibodies will be essential in order to confirm the true relationship.
